# Vitamin D receptor gene polymorphisms in breast and renal cancer: Current state and future approaches

**DOI:** 10.3892/ijo.2013.2204

**Published:** 2013-12-02

**Authors:** MOHAMMED I. KHAN, ZOFIA F. BIELECKA, MOHAMMAD Z. NAJM, EWA BARTNIK, JERZY S. CZARNECKI, ANNA M. CZARNECKA, CEZARY SZCZYLIK

**Affiliations:** 1 Molecular Oncology Laboratory, Clinic of Oncology, Military Institute of Medicine, 04-141 Warsaw;; 2 Institute of Genetics and Biotechnology, Faculty of Biology, University of Warsaw, 02-106 Warsaw;; 3 Institute of Biochemistry and Biophysics, Polish Academy of Sciences, 02-106 Warsaw, Poland;; 4 Department of Biochemistry, Jamia Hamdard University, New Delhi 110 062, India;; 5 Department of Knowledge Management, Faculty of Management, University of Lodz, 90-237 Lodz, Poland

**Keywords:** vitamin D, vitamin D receptor gene polymorphisms, breast cancer, renal cancer

## Abstract

Cancer is a major health problem and cause of death worldwide that accounted for 7.6 million deaths in 2008, which is projected to continue rising with an estimated 13.1 million deaths in 2030 according to WHO. Breast cancer is the leading cause of cancer-based death among women around the world and its incidence is increasing annually with a similar tendency. In contrast, renal cell carcinoma accounts for only 3% of total human malignancies but it is still the most common type of urological cancer with a high prevalence in elderly men (>60 years of age). There are several factors linked with the development of renal cell cancer only, while others are connected only with breast cancer. Genetic risk factors and smoking are the factors which contribute to carcinogenesis in general. Some evidence exists indicating that vitamin D receptor (VDR) gene polymorphisms are associated with both breast and renal cancer; therefore, we put forward the hypothesis that polymorphisms in the VDR gene may influence both the occurrence risks of these cancers and their prognosis. However, the relationship between VDR polymorphisms and these two specific cancers remains a controversial hypothesis, and consequently needs further confirmation via clinical research together with genetic investigations. Here, we aimed to assess the correlation between the different alleles of VDR gene polymorphisms and renal cell cancer and breast cancer risks separately through a systematic review of the present literature. In contrast, this analysis has revealed that some VDR gene polymorphisms, such as: Bsm1, poly(A), Taq1, Apa1, are to some extent associated with breast cancer risk. Other polymorphisms were found to be significantly associated with renal cell cancer. Namely, they were Fok1, Bsm1, Taq1 and Apa1, which encode proteins participating mainly in proliferation, apoptosis and cell cycle regulation. However, data concerning renal cancer are not sufficient to firmly establish the VDR gene polymorphism association.

## Contents

1. Introduction
2. Vitamin D and cancer
3. Vitamin D receptor
4. Common VDR polymorphisms and their associated alleles
5. Exploration of VDR polymorphisms
6. Ethnic variation is crucial in predicting effects of VDR gene polymorphisms
7. Vitamin D and breast cancer
8. Vitamin D and renal cancer
9. Search methods for the meta-analysis
10. Results of the meta-analysis
11. Concluding remarks

## 1. Introduction

Cancer is the leading cause of death worldwide, and accounted for 7.6 million deaths in 2008. The incidence of cancer continues to rise with an estimated 13.1 million deaths in 2030 (1). Breast cancer is a common disease and the leading cause of death among women around the world, with its incidence is increasing annually. It is estimated that 1 in 12 British women and 1 in 8 American women will develop breast cancer at some time in her life, the overall death ratio among women with this disease has been established at 20%, and the expected time of survival, at 5 years after the diagnosis (2). There are several constant risk factors for breast cancer, namely age, gender, density of breast tissue, benign breast conditions, early menarche, late menopause, previous chest exposure to radiation, exposure to diethylstilbestrol and genetic risk factors (BRCA-1 and BRCA-2) (3,4). As for the risk factors that may differ across patients, they include nulliparity or the first pregnancy after the age of 30, postmenopausal hormone treatment, combined use of estrogen and progesterone, breast feeding, alcohol consumption, postmenopausal obesity and insufficient physical activity (90). When renal cancer is taken into consideration, it accounts for 3% of total human malignancies and is the most common type of heterogeneous urological cancer with high prevalence in elderly men (>60 years old) (5). Because of the high mortality level due to metastasis (Czech Republic, Poland) renal cancer is considered to be one of the most important urological cancers in Central and Eastern Europe (6,89). There are several risk factors linked to the development of renal cancer, such as obesity, smoking, high blood pressure, ethnicity, age and family history or genetic risk factors [the most common one being the von Hippel-Lindau (VHL) syndrome] (7). Therefore, in this review we aimed to assess the correlation between the different alleles of VDR gene polymorphisms and renal cell cancer and breast cancer risks separately, through a systematic review of the present literature.

## 2. Vitamin D and cancer

**Figure 1.**
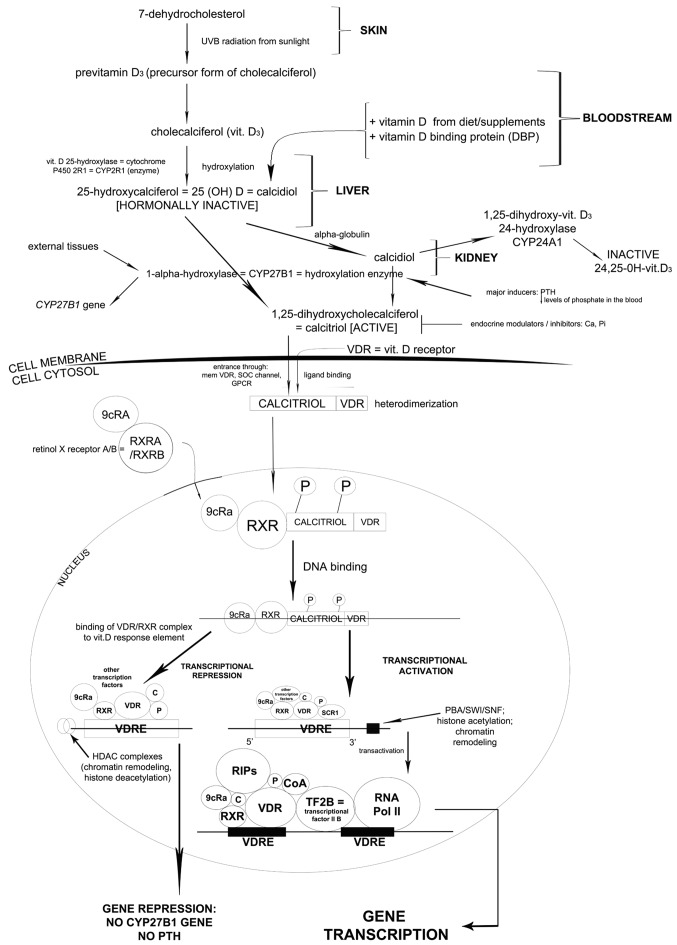
7-Dehydrocholesterol undergoes specific alterations in the skin, liver and kidney. As a result calcitriol is formed and enters the cell cytosol through specific channels together with vitamin D receptor (VDR). After heterodimerization, the complex enters the nucleus and as a result, a multi-component complex is formed (phosphorylated calcitriol-VDR complex, retinol X receptor, 9cRa transcription factor) which subsequently binds to DNA. In the presence of HDAC complexes and other transcription factors, CYP27B1 gene responsible for parathormone production is repressed. However, in the presence of PBA/SWI/SNF complex other compounds are added (regulators of interaction, transcriptional factor IIB and most important of all, RNA polymerase II). As a consequence, CYP27B1 transcription occurs. The protein encoded by this gene localizes to the inner mitochondrial membrane where it hydroxylates 25-hydroxyvitamin D3 at the 1α position. This reaction synthesizes 1α,25-dihydroxyvitamin D3, the active form of vitamin D3, which binds to the VDR.

There are two major forms of vitamin D: D2 [calciferol or 1,24-dihydroxyvitamin D(2)] and D3 [calcitriol or 1,25-dihydroxyvitamin D(3)]. Anticancer properties have been attributed primarily to vitamin D3 (8). Vitamin D3 binds to the vitamin D receptor (VDR). This ligand-receptor complex regulates transcription of >60 genes involved in anti-proliferative, pro-differentiating, anti-metastatic and pro-apoptotic effects on cells and the cell cycle (Fig. 1: vitamin D conversion pathways) (9–12). In the mammary gland, vitamin D3 regulates calcium transport during lactation and acts together with mammary cell differentiation hormones and milk protein production (13). Laboratory studies have also demonstrated that vitamin D3 and its analogues inhibit cell proliferation and promote apoptosis in cancer cells in culture (14–18). These findings have led to the development of vitamin D analogues that could serve as potential new therapeutic agents in breast cancer treatment in humans. The observation of a reduced risk of breast cancer among women with high vitamin D status also supports the hypothesis that vitamin D plays a crucial role in cancer development (19–21). However, breast cancer is also known to be strongly influenced by the hormonal milieu and mutations in genes involved in hormone metabolism. The kidney has a unique function in mineral homeostasis especially for calcium and phosphorous. The vitamin D-endocrine system plays a key role in controlling the reabsorption of calcium by the kidney (22,23). The kidney is the major site for synthesis of vitamin D3 (24). Other vitamin D3-dependent proteins and the VDR that are important in calcium reabsorption are also expressed in the kidney (25,26). All in all, biological and epidemiological data suggest that vitamin D3 levels influence the development of renal cancer (27–29).

## 3. Vitamin D receptor

**Figure 2.**
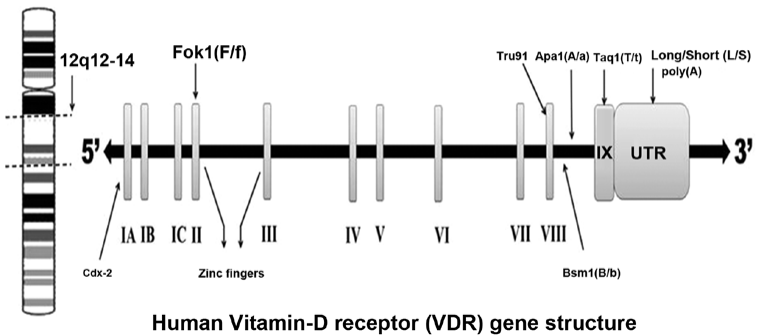
Human vitamin D receptor (VDR) gene polymorphic sites.

The VDR was discovered in 1969 (30). The VDR receptor gene is located on the long arm of chromosome 12 (12q12–14) with at least five promoter regions, and is composed of at least 11 exons that span 60 kb of DNA (31,32). The first exon is not translated (33), while exons 2–8 encode the VDR protein (Fig. 2). Polymorphisms of the VDR gene have been associated with several forms of cancer and other chronic diseases (30). The associations between the VDR gene polymorphisms and breast and renal cancers have been investigated by a number of studies (20,29,34–36). It has been postulated that the VDR gene polymorphisms may influence both the risk of cancer occurrence and its prognosis. The purpose of this review is to analyze the association of some common VDR gene polymorphisms, such as Taq1, Fok1, Apa1, Bsm1, Cdx2, poly(A) and Bgl1, with breast and renal cancers.

## 4. Common VDR polymorphisms and their associated alleles

Most studies on the VDR gene polymorphisms association with breast and renal cancers have focused on seven types of polymorphisms: Fok1 polymorphism in exon II (37), Bsm1 (38) and Apa1 (39) in intron VIII, Cdx2 (40) in exon I, Taq1 (41) in exon IX, Tru91 (42) in intron VIII, and the poly(A) (43) mono-nucleotide repeat in the 3’-untranslated region (3’-UTR) section of the gene. Individuals are generally classified as tt, Tt or TT for Taq1 polymorphism (T allele: absence of Taq1 restriction site; t allele: presence of Taq1 restriction site). The Fok1 polymorphism is located near the 5’-UTR region of the gene. The f allele for the Fok1 polymorphism shows less transcriptional activity than the F allele (44,45). All other polymorphisms have been found close to the 3’-UTR region of the gene (46). The polymorphisms in the 3’-UTR region of the gene appear to be in linkage disequilibrium (LD), and the allele frequencies for these polymorphisms appear to vary across populations (47). Individuals with the Bsm1 polymorphism are classified as bb, Bb, BB genotypes. With respect to poly(A), a bi-allelic polymorphism, an individual can be classified as: S (short, with <18 A’s) and L (long, with >168 A’s) poly(A) stretches (45). The individuals associated with Apa1 polymorphism are classified as aa, Aa and AA genotypes. In general, most of the polymorphisms in the VDR gene are located in regulatory areas rather than in the coding exons (48).

## 5. Exploration of VDR polymorphisms

The VDR polymorphisms that have been studied using the restriction fragment length polymorphism (RFLP) technique involve Apa1 (39), Bsm1 (38) and Taq1 (41) restriction polymorphisms at the 3’-end of the VDR gene. The Fok1 polymorphism occurs due to a thymine/cytosine (T/C) change (44). It basically alters an ACG codon to an ATG codon located ten base pairs upstream from the translation start codon, which results in the formation of an additional start codon. If protein translation starts from this altered site, a larger VDR protein with three additional amino acids will be formed (49). Taq1 is a substitution of nucleotide ATT for ATC in exon IX, leading to a synonymous change at codon 252 (isoleucine) (50,51). Although Bsm1 and Apa1 are considered as silent single nucleotide polymorphisms (SNPs) that do not change the sequence of coding amino acids like in Fok1 (52), they may influence gene expression through regulation of mRNA stability. The Cdx2 polymorphism is a guanine (G) to adenine (A) alteration in the promoter region of the VDR gene, specifically at the binding site for an intestinal specific transcription factor known as Cdx2 (53). The A allele binds to the Cdx2 transcription factor with a higher affinity, and yields increased transcriptional activity (40). As a consequence, the A allele may result in a higher VDR expression in the intestine, and therefore in an increased bone mineral density (BMD) through a better intestinal absorption of calcium (35,40,54). The poly(A) polymorphism occurs in the 3’-UTR region of the VDR gene, which is characterized by a variable number of tandem repeats (VNTR) (43). Finally, two polymorphisms, namely A-1012G and Tru91, have been poorly investigated. The A-1012G polymorphism consists in a substitution of A for G (55). The Tru91 is a G (U allele) to A (u allele) polymorphism in the VDR gene (42).

## 6. Ethnic variation is crucial in predicting effects of VDR gene polymorphisms

Genetic studies based on different ethnic backgrounds provide excellent opportunities to link molecular insight with epidemiological data (35). Variation in DNA sequences occurs frequently in the population, and has a substantial biological impact on the development of certain diseases, including cancer. The Cdx2 polymorphism was first discovered in the Japanese (40), and has recently been studied in a Caucasian population by Fang *et al* (54). However, the study revealed that the Cdx2 polymorphism A allele occurs more commonly in African (74%) and Asian (43%) populations than among the Caucasians (19%). The data obtained from their research has shown that the f allele for the Fok1 polymorphism occurs less frequently among the Africans (24%) than among the Caucasians (34%) and the Asians (51%), while the frequency of the B allele for Bsm1 is much lower in the Asian (7%) population than among the Caucasians (42%) and the Africans (36%) (35). On the other hand, the Apa1 A allele is exhibited at a higher frequency in the Asian population (74%) than among the Caucasians (44%) and Africans (31%).

The LD describes the co-occurrence of the alleles of adjacent polymorphisms. In consequence, the presence of one type of polymorphism may serve as indication of the presence of another polymorphism that is linked to it. It is noticeable due to a very slight recombination that has occurred between them during evolution. LD is present in the case of both Taq1 and poly(A), since they occur in similar ratios in different ethnic groups, with a lower percentage of the Taq1 T allele among Asians (8%) compared to Caucasians (43%) and Africans (31%); similar results have been observed for the poly(A) S allele, which is less frequent among the Asians (12%) than among the Caucasians (41%) and the Africans (29%) (52). In contrast to the Bsm-Apa-Taq haplotypes, haplotype 3 (bAT) is common among the African population (59%), while haplotype 1 (baT) and haplotype 2 (BAt) are mostly observed in Asian (75%) and Caucasian populations (39%) respectively (35).

## 7. Vitamin D and breast cancer

**Figure 3.**
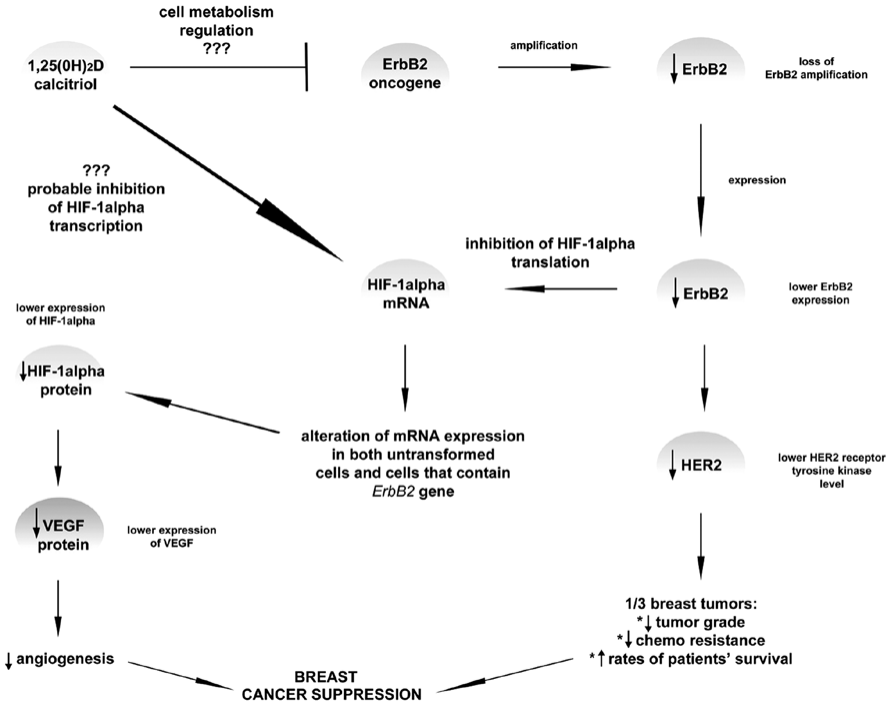
It is known that vitamin D plays a crucial role in reducing the risk of breast cancer development, basically when the prevention of angiogenesis is taken into account. VEGF is a potent angiogenic factor which was shown in several publications to limit the vessels production due to the 1,25(0H)2D impact via the hypoxia-inducible factor-1α (HIF-α). It has been proposed that dihydroxyvitamin D does not directly affect VEGF expression. In some of the research, ErbB, a gene responsible for breast cancer invasiveness, has been shown to increase HER2 expression. Amplification or overexpression of this gene is perceived as one of the major factors contributing to pathogenesis and progression of certain aggressive types of breast cancer and in recent years it has become a significant biomarker and target of therapy for this disease. However, it was suggested that lower ErbB2 expression leads to the inhibition of HIF-α translation which subsequently leads probably to lower VEGF and HER2 expression. This means that higher rates of patient survival may be achieved. Currently, the role of calcitriol in connection with HIF-1α expression level is still not fully confirmed, however, inhibition mechanism appears to be the most probable among other theory. Moreover, ErbB2 is also probably inhibited by calcitriol; yet, the mechanism of such action is still unknown.

Currently, *in vitro*, preclinical and clinical findings support the hypothesis that low levels of vitamin D are linked to an increased risk of breast cancer (56). Anti-carcinogenic effects of vitamin D are mediated via the estrogen pathway by downregulation of the estrogen receptor (ER), which inhibits cancer cell proliferation, induces cell apoptosis, and prevents carcinogenesis *in vitro* and in animal models (57–59). On the other hand, epidemiological data also show that vitamin D supplementation in invasive breast cancer patients did not reduce breast cancer incidence in one trial conducted on post-menopausal women (60). These contradictory findings could be related to tumor heterogeneity, which suggests that effects of vitamin D may only be exhibited in specific subtypes of breast cancer. Therefore, additional functional experiments with vitamin D supplementation should be conducted on specific breast cancer subtypes and polymorphisms in the VDR gene and other gene variants (Fig. 3).

## 8. Vitamin D and renal cancer

**Figure 4.**
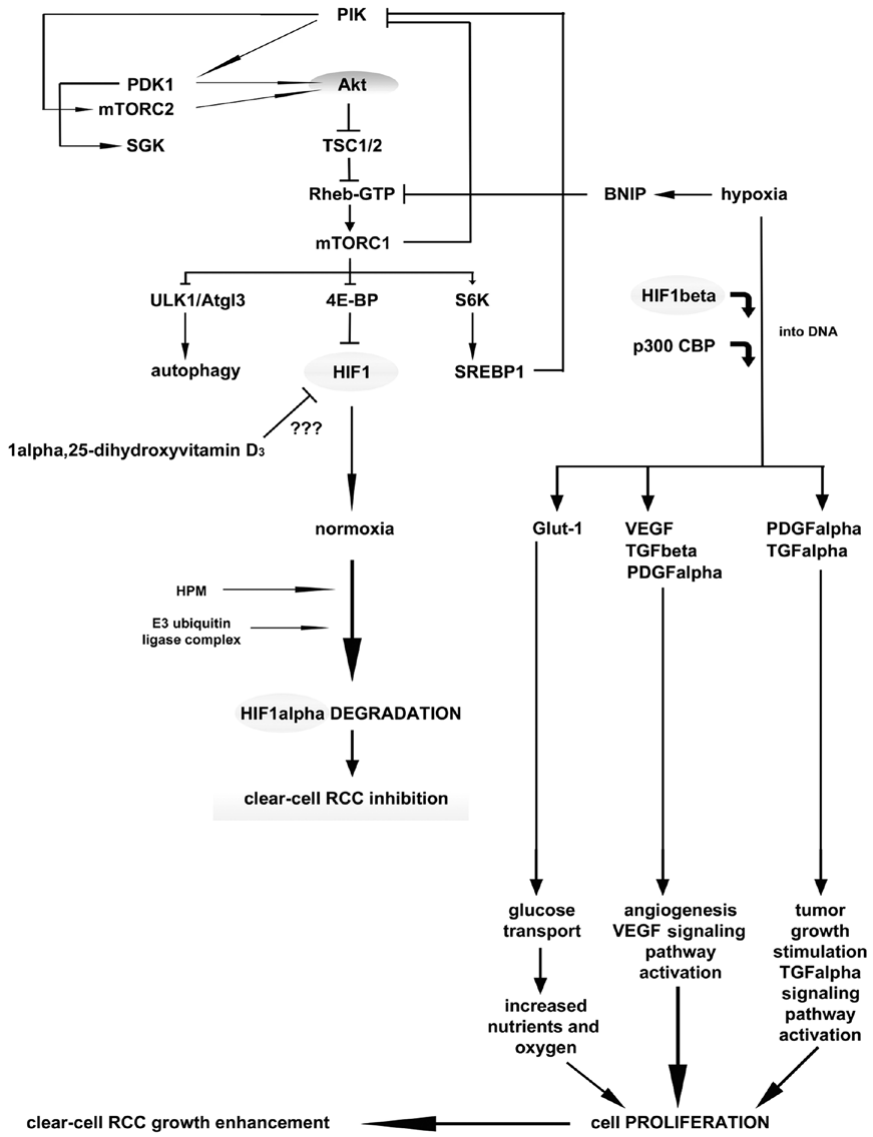
RCC of clear cell type is the most common form of renal cancer. One of the factors which inhibits hypoxia in RCC is hypoxia-inducible factor-1α (HIF-α). It is unstable in normoxia with its half-life of <10 min. Hypoxia is a widely known mechanism of angiogenesis regulation. As a result of adaptation of cancer cells to hypoxia conditions, cancer cells in RCC gain resistance both to chemotherapy and radiotherapy. HIF-1α enhances cellular oxygen tension which in consequence leads to normoxia and subsequently HIF-1α degradation occurs. HIF transcription complex has been shown to induce the expression of among others, VEGF and PDGFα, which results in cell proliferation and tumor growth. However, the problem of the impact of hypoxia is a matter of different research and this article will not focus on this aspect of RCC. The other vital ATP consumer that contributes to hypoxia inhibition in mRNA translation process. Both mammalian target of rapamycin and pancreatic kinase (Eip2A) with the help of other transcription factors presented in the figure, take part in hypoxia regulation. The first one, which is a large serine/threonine protein kinase is also involved in homeostasis regulation as it controls catabolic and anabolic processes such as oxygen availability and growth factor signaling. mTOR complex 1 (mTORC1) is also responsible for its downstream actions which are autophagy regulation, protein synthesis and metabolic pathways control. Still, it is not clear how exactly vitamin D3 may inhibit the activation of HIF-1α. One of the hypothesis is that it participates in the regulation of blood vessel supply to the tumor, regulated by hypoxia/normoxia state.

In the kidney, the major site of vitamin D3 formation, metabolism, activity and calcium homeostasis under physiological conditions is the renal proximal tubule (61). Epidemiological data suggest that vitamin D3, obtained either from dietary intake or as a result of the body’s exposure to ultraviolet light, is inversely correlated with renal cancer risk (62,63). Vitamin D3 serum concentrations have been found to be significantly decreased in patients with renal cancer compared to the control population (28,64). However, the exact role of anti-carcinogenic mechanism of vitamin D3 has not been studied widely nor is it completely understood. Nevertheless, it can be postulated that vitamin D3 impedes carcinogenesis via the VDR, and stimulates cell differentiation by inhibiting cell proliferation, inducing apoptosis and suppressing invasiveness, angiogenesis and metastasis (Fig. 4) (63,65,66). The kidney is the most vital organ for vitamin D metabolism and calcium homeostasis, though there is still scarcity of data on the association between dietary vitamin D3, VDR gene polymorphism and renal cancer etiology.

## 9. Search methods for the meta-analysis

### 

#### Strategies to recover the published data

Most epidemiological studies of breast and renal cancers have been concentrated on seven polymorphisms of the VDR gene that are potentially important for the cancer etiology. We performed a meta-analysis of the published literature using the PubMed Central^®^ (PMC), a biomedical and life sciences journal literature from the National Library of Medicine (NLM). The search was conducted using literature data from January, 1997 until November, 2012. The search employed combinations of keywords ‘VDR gene polymorphisms’ with ‘breast cancer’ and ‘renal cell cancer’, as well as of the keywords: Fok1, Bsm1, Taq1, Apa1, poly(A), Cdx2, Bgl1, A-1012G and Tru91 with ‘breast cancer’ and ‘renal cell cancer’. In the case of breast cancer, 52 results were generated, while only 12 results were found in the case of VDR polymorphisms and renal cancer.

## 10. Results of the meta-analysis

### Fok1 polymorphism vs. breast cancer and renal cancer

#### Fok1 polymorphism and breast cancer

Most of the case control studies performed on different ethnic populations did not show that the risk of breast cancer was associated with the Fok1 polymorphism (34,67–70). An analysis conducted recently on Chinese women also supported this statement (70). The Fok1 polymorphism was not found to be associated with breast cancer when analyzed separately. However, Fok1 did modulate the increased risk of other VDR genotypes (bb/LL genotype Bsm1 b allele, poly(A) L allele) together with the bb/LL genotype, which resulted in an increased breast cancer risk (68). In one population-based case control study, no effect of the concentration of 25(OH)D serum and VDR genotype was observed, and none of the analyzed polymorphisms were correlated with breast cancer risk (34). Another study conducted among the UK Caucasian population showed similar results, with no association of Fok1 with breast cancer (67). In the investigations conducted on 500 breast cancer cases in post-menopausal women and 500 controls matched by age, ethnicity and blood collection date, similar results were obtained (71). Others have also obtained the same result in their analysis (20). Curran *et al* (72) have shown in the Australian population that VDR initiation codon Fok1 polymorphism is not associated with breast cancer occurrence. Interestingly, a significantly increased risk of breast cancer was observed in one large study (1,234 cases and 1,676 controls) among carriers of the ff genotype of Fok1 (multivariate OR=1.34) compared with those with the FF genotype (73). In that study, the Fok1 association was influenced by the menopausal status, estrogen, the progesterone receptor status of the tumors, and plasma levels of 25(OH)D or 1,25(OH)2D3. Furthermore, a meta-analysis of 21 case-control studies with Fok1, Bsm1, Apa1 and Taq1 polymorphisms has shown that the Fok1 polymorphism was associated with an increased risk of breast cancer development (ff vs. FF: OR=1.15; 95% CI, 1.03–1.28; the recessive model ff vs. Ff + FF: OR=1.14; 95% CI, 1.03–1.26) (74), while in a sub-analysis a significant association was found between the Fok1 polymorphism and breast cancer in the European population. Current meta-analysis has shown that Fok1 may serve as a diagnostic biomarker for breast cancer susceptibility especially in the European population. Taking all these factors into consideration, the above studies showed an uncertain association of Fok1 with breast cancer. Further studies are still needed to clarify the observations based on the Fok1 polymorphism association with that cancer. Table I summarizes the reference analysis of the Fok1 polymorphism association with breast cancer.

#### Fok1 polymorphism and renal cancer

The association of Fok1 with renal cell cancer has not been studied widely. The scarce published data comprise the analysis of Fok1 and its role in renal cell cancer development; however, a recent study yielded contradictory results, it was conducted in Central and Eastern Europe, and showed that the subjects over 60 years of age who were carriers of the f alleles in the Fok1 SNP had reduced renal cancer risk compared to the subjects with the FF genotype (75). However, recent research on the North Indian population has shown contradictory findings, in which an increased number of the Fok1 polymorphism alleles was linked to a high risk of renal cell cancer considering other risk factors, such as: hypertension, smoking and improper body mass index (BMI) (76). These results show the association of Fok1 polymorphism with renal cancer. However, the result varies depending on the ethnic background, so further research is needed for confirmation. Table II summarizes the reference analysis of the Fok1 polymorphism association with renal cancer.

### Bsm1 polymorphism vs. breast cancer and renal cancer

#### Bsm1 polymorphism and breast cancer

A strong association has been found between the bb genotype of the Bsm1 VDR polymorphism and increased breast cancer incidence. A great number of research studies have been published to support this statement, but some conclusions differed across the individual authors and ethnic groups. Research based on UK Caucasian women has shown a 1.8-fold increase of cancer risk for the bb genotype individuals (OR=1.79) (77). In addition, >70% of seven commonly used breast cancer cell lines have been found to possess the risky bb genotype. Guy *et al* (68) have further confirmed that Bsm1 is associated with a high risk of breast cancer development, and the likelihood of developing breast cancer is nearly twice higher for a woman with the bb genotype than for a woman with BB or Bb genotype (bb genotype OR=1.92; BB genotype OR=1.00; and Bb genotype OR=1.00). Similar results have been reported for the Caucasian women, but not for the African-American women, where this polymorphism increased the risk of breast cancer development in postmenopausal carriers of the bb genotype of Bsm1 (OR=1.53) (78). It is also worth noticing that the smoking status modified this association of Bsm1 genotype and breast cancer risk. Premenopausal Caucasian women who had reported smoking at least once had an increased risk of breast cancer. However, there was no such association in case of the smoking habit among postmenopausal Caucasian women, premenopausal African-American women or postmenopausal African-American women. The respective association between Bsm1 genotypes and breast cancer risk does not vary significantly with oral contraceptive use, hormone replacement therapy, or BMI (78).

Another study has reported that the Bsm1 polymorphism was in LD with the poly(A) sequence in the 3’-non-translated region, and the ‘L’ poly(A) variant was also associated with breast cancer susceptibility similarly to Bsm1 (bb vs. BB genotype OR=2.32) (67). The study by Lowe *et al* (12) also reported that low levels of circulating 25(OH)D (<50 nM) occurring in association with the bb Bsm1 VDR genotype may increase the risk of breast cancer more than levels of 25(OH)D >50 nM in either the BB or the Bb genotype. Another study shows that VDR polymorphism Bsm1 distributions in the case group and in the control group of patients did not exhibit any statistical difference. On the other hand, the metastatic cancer group with prevalence of the bb genotype (14/38; 37%) was twice as large as the corresponding percentage of control subjects, whereas the percentage of BB women with metastases was half lower than in the control group (2/38; 5%) (79). Moreover, homozygous bb women have been shown to develop metastases four times more often than BB women.

Ingles *et al* (69) have reported that when the bb genotype was compared with the Bb and BB genotypes separately, it was shown to have a 1.6- and 2.2-fold increased breast cancer risk, respectively. In Taiwanese women, the Bsm1 B allele was also associated with an increased breast cancer risk (80). The data showed that 79% of the breast cancer group and 76% of the benign breast tumor group had the bb genotype compared to 91% of the control population. Only 9% of the control population had the B allele, in contrast to the breast cancer group (21%) and the benign breast tumor group (24%), which supports the B allele association with an increased breast cancer risk. However, a number of studies have also yielded conflicting data on association of the Bsm1 VDR polymorphism with breast cancer risk. Two independent case-control studies conducted on the same population have shown that the Bsm1 Bb + bb genotype is not associated with an increased risk of breast cancer in the French Canadian population (OR=1.22) without interaction with family history (81). Similar results have been obtained by another group, who has shown the absence of any significant association between Bsm1 polymorphisms and breast cancer development on a small group (78 patients) of the Turkish population (82). In Caucasian women, no association was found between polymorphisms in Bsm1 and breast cancer risk, OR=0.93 for BB vs. bb (73). However, these results suggest that the VDR polymorphism may be a mediator of cancer risk, and could be a target for cancer prevention efforts. Some results showed that breast cancer incidence was not associated with any genotype (71). However, women with the Bsm1 bb genotype that consumed >902 mg/day of calcium had lower susceptibility to breast cancer than those with the Bb or BB genotype (OR=0.61). In conclusion, the Bsm1 bb genotype is associated with breast cancer risk in Caucasian and African-American women, despite the fact that this association is influenced by other factors, such as smoking. Table III summarizes the reference analysis of the Bsm1 polymorphism association with breast cancer.

#### Bsm1 polymorphism and renal cancer

Although many detailed studies have been conducted in the field of renal cell cancer, more data are undoubtedly required to determine the importance of the Bsm1 polymorphism association with renal cell cancer development. Only a few studies have been published that showed its connection with renal cancer. A previously conducted study in the Japanese population showed that the Bsm1 variant did not have any statistically significant effect on the risk of renal cancer (36). However, when the BB genotype of the Bsm1 gene polymorphism was analyzed in Central and Eastern Europe, an individual with a positive family history of cancer had a lower renal cancer risk compared to an individual with the bb allele (75). Similar results have been obtained for the Indian population, showing that Bsm1 polymorphism (bb genotype) can significantly modify the risk of renal cancer (76). All these findings show the potential of Bsm1 in predicting the risk of developing renal cell cancer, though further studies taking into consideration other ethnic groups are needed for confirmation. Table IV summarizes the reference analysis of the Bsm1 polymorphism association with renal cancer.

### Taq1 polymorphism vs. breast cancer and renal cancer

#### Taq1 polymorphism and breast cancer

A large number of studies have shown the absence of any association between the Taq1 VDR polymorphism and breast cancer susceptibility. A population-based case-control study (PCCS) has revealed that high sun exposure reduces the risk of advanced breast cancer development among women with light constitutive skin pigmentation (OR=0.53), and that this association does not depend on the Taq1 VDR genotype (20). Similar results showing no significant association between breast cancer risk and the Taq1 genotype have been obtained in different ethnic backgrounds (71,82–84). In the Taiwanese population, where low frequency of breast cancer was found, no association of Taq1 with breast cancer has been established (80). On the other hand, a tendency for a decreased risk of breast cancer has been observed for the Taq1 T allele (OR=0.68), but proper estimation of the potential impact of Taq1 polymorphism on breast cancer was impossible to perform (85). A number of Taq1 analyses comprising the Taq1 polymorphism in combination with dietary factors have also been reported. Women with the TT genotype who consumed >902 mg total calcium per day exhibited lower breast cancer risk compared to genotypes Tt or tt (71). The Taq1 polymorphism association with a significantly increased risk of estrogen receptor positive tumors (OR=1.18) for t allele carriers compared to non-carriers (OR=0.88) has also been reported (34,52). In a case-control study (CCS) of Australian women, a tendency for the Taq1 T allele association with an ∼1.5-fold increased breast cancer risk has been found (72). Finally, for patients with the TT genotype, a significantly increased risk (OR=1.8) for lymph node metastasis has been reported. Furthermore, among patients with the tt genotype, a tendency towards prolonged survival has been noted among ER-positive, tamoxifen-treated patients (86). In conclusion, polymorphisms in the VDR gene may influence tumor progression and tamoxifen treatment response in early-onset breast carcinomas (86). It appears that the T allele is a ‘risk allele’, while the t allele is a ‘protective allele’. Table V summarizes the reference analysis of the Taq1 polymorphism association with breast cancer.

#### Taq1 polymorphism and renal cancer

There is scarce data concerning the effect of the Taq1 polymorphism on renal cancer occurrence and outcome. A lower risk of renal cancer has been observed among individuals with the t allele in the Taq1 gene polymorphism in the Central and Eastern European population (75), and this polymorphism was not differentiated by the tumor stage or grade. Previously, a study of the Japanese population has also been carried out, revealing that: i) the TT genotype was statistically more frequent among renal cancer patients (80.4%); ii) TT frequency was higher compared to rapid tumor growth type group (92.1%) and slow tumor growth type group (73.4%) (87). All these analyses have shown that the T allele is associated with determining the risk of developing renal cancer. On the contrary, a recent study of the Japanese population has shown contradictory results, and has not found any statistical difference (36). Therefore, further research is needed on the basis of cancer stage to define or confirm the role of the T allele in renal cancer risk. Table VI summarizes the reference analysis of the Taq1 polymorphism association with renal cancer.

### Apa1 polymorphism vs. breast cancer and renal cancer

#### Apa1 polymorphism and breast cancer

The association of Apa1 with breast cancer has not been widely studied. Different approaches towards the Apa1 association analysis have been used, and some studies have revealed that the Aa and aa genotypes were significantly associated with an increased breast cancer risk (OR=1.56) (72). Contradictory results have been obtained among the Taiwanese population (80). Those results have shown that the AA genotype has a tendency to increase cancer risk, whereas decreasing copies of the A allele have been found to be associated with a reduced cancer risk (OR=0.333 for the Aa genotype and OR=0.515 for the aa genotype). In other studies conducted on the Finnish population, a statistically significant difference was observed in the Apa1 genotype distribution between the cases and the controls (85). Women with the VDR variant ‘a’ genotypes had a decreased risk of breast cancer (OR=0.73) compared with women carrying the AA genotype. If the family history of breast cancer was taken into consideration, this association became strong (OR=0.14). The results also suggest that the AA genotype is more common among women with breast cancer, whereas the lowest risk of breast cancer is found in women with the aa genotype (OR=0.03) (85). On the other hand, other research groups have not found such an association (71,84). In conclusion, the Apa1 allele association has not been analyzed widely in different racial groups, but in most of the studies it has been found to be linked with breast cancer risk. Table VII summarizes the reference analysis of the Apa1 polymorphism association with breast cancer.

#### Apa1 polymorphism and renal cancer

Only one study on the Japanese population has analysed the association between renal cancer and the AA genotype of the Apa1 polymorphism in the VDR gene (36). The frequency of the AA genotype has been found to be significantly higher in renal cancer patients (17.0%) than in the control group (7.3%). More than half (52.2%) of the patients with the AA genotype had distant or lymph node metastases compared to the Aa + aa genotypes (6.3%). Moreover, patients with the AA genotype were found to have poor survival rates compared to patients with other genotypes (Aa + aa). In conclusion, these findings suggest that the AA genotype may influence disease progression and prognosis of renal cancer; however, these results are limited to a certain ethnic background. Thus, further research concerning the Apa1 polymorphism for other populations are necessary. Table VIII summarizes the reference analysis of the Apa1 polymorphism association with renal cancer.

### Poly(A) polymorphism vs. breast cancer and renal cancer

#### Poly(A) polymorphism and breast cancer

The claim concerning the presence of an association between the poly(A) polymorphism and breast cancer occurrence is controversial. In a recent study carried out on the Swedish population, women carrying two short poly(A) alleles (SS genotype) were found to have an increased risk of breast cancer incidence (OR=1.26) (88). There was a statistically significant interaction between the VDR genotype and parity; women with two short alleles (SS) had a halved risk of breast cancer, irrespective of the parity, compared with nulliparous women with two long alleles (LL). On the other hand, homozygosity for the long VDR allele (LL) was associated with a more advanced clinical stage at diagnosis (88). With respect to a previous study, when the LL genotype was compared to the S genotype with an increased number of short poly(A) alleles, a higher risk of developing cancer was associated with the number of S variants (OR=1.5 for the SL genotype and OR=3.2 for the SS genotype) (69). Some other studies carried out on Caucasian women also suggested the association of poly(A) with breast cancer (67). The poly(A) variant L was associated with an increase of breast cancer risk because, poly(A) was in LD with the Bsm1 polymorphism (67). Similar results have also been obtained by another group (OR=1.94 for LL vs. SS genotype) (68). Some studies have also yielded contradictory results for the poly(A) genotype (71). However, women with the poly(A) (LL) repeat who consumed more than 902 mg/day of calcium exhibited decreased risk of breast cancer incidence (71). On the other hand, breast cancer and the poly(A) genotype were not found to be associated in a recent study on a group of Caucasian and African-American women (78). It has been found that the association of poly(A) with breast cancer can be modified by the smoking status, but it did not significantly vary with oral contraceptive use, hormone replacement therapy, or BMI (78). In conclusion, the role of poly(A) is still controversial, but the variant LL allele is associated with breast cancer risk, though further confirmation is undoubtedly needed. Table IX summarizes the reference analysis of the poly(A) polymorphism association with breast cancer.

#### Poly(A) polymorphism and renal cancer

No specific data basis has been found so far to demonstrate a direct association of poly(A) with the risk of renal cancer. However, some studies have found a correlation between Taq1 and the poly(A) polymorphism, which are in LD with each other in the Asian, Caucasian and African populations (35). A lower risk of renal cancer has been observed for the t allele of the Taq1 gene polymorphism (75,87). All these studies examined solely the role of the Taq1 polymorphism on its own. However, it is feasible that poly(A) may show an important role in renal cancer development when studied together with the Taq1 polymorphism.

### Cdx2 and Bgl1 polymorphism vs. breast cancer and renal cancer

#### Cdx2 and Bgl1 polymorphism and breast cancer

Undoubtedly, not enough work has been done on the potential functional association between Cdx, Bgl1 and breast cancer risk. No LD has been found between the Cdx2 and other VDR genes (Fok1, Taq1 and VDR-5132) with respect to the breast cancer risk (34). In a PCCS carried out on various racial groups, such as Hispanic, African-American and non-Hispanic Caucasians, similar results were obtained for the linkage of Bgl1 to breast cancer risk (20). High exposure to sunlight was linked with reduced risk of breast cancer among women with light constitutive skin pigmentation (OR=0.53), but not among women with medium or dark pigmentation (20). In conclusion, the data on VDR Cdx and Bgl1 polymorphism are still limited.

#### Cdx2 and Bgl1 polymorphism and renal cancer

There are no published data so far that would describe the role of Cdx2 and Bgl1 with regard to the risk of developing renal cell cancer. Table IX summarizes the reference analysis of the Cdx2 and Bgl1 polymorphism association with breast cancer.

## 11. Concluding remarks

In this study we have analyzed the polymorphisms in the VDR gene occurring most commonly in breast cancer and renal cancer. Based on the set of data collected from different studies performed on different ethnic populations, it is not possible to make an authoritative declaration on the role of VDR polymorphisms in breast and renal cancer development and prognosis. However, some polymorphisms have been found to be strongly associated with breast cancer and some with renal cancer. Understanding the role of VDR polymorphisms provides mechanistic insight and may facilitate the development of new preventive strategies for breast and renal cancer.

## Figures and Tables

**Table I.** Fok1 and breast cancer.

Population studied	Study type	Polymorphism	Cases/controls	Results	Refs.
Latinas	CCS	Fok1, Bsm1, poly(A)	143/300	No association	(69)
Caucasian	CCS	Fok1, Bsm1	313/410	No association	(77)
Caucasian	CCS	Fok1, Bsm1, poly(A)	398/427	No association	(68)
Caucasian	CCS	Fok1, Bsm1, poly(A)	181/241	No association	(67)
German	PCCS	Fok1, Taq1, Cdx2, VDR-5132	1408/2612	No association	(34)
Caucasian	NCCS	Fok1, Bsm1, Taq1, Apa1, poly(A)	500/500	No association	(71)
Australian	CCS	Fok1, Taq1, Apa1	135/410	No association	(72)
USA	CCS	Fok1	1234/1676	The ff genotype associated with breast cancer risk	(73)
French Canadian	CCS	Fok1, Bsm1	Two independent studies (225/463 and 622/974)	The ff genotype linked to higher breast cancer risk	(78)
Hispanic, non-Hispanic	CCS	Fok1, Taq1, Bgl1	814/910	No association	(20)
Chinese	PCCS	Fok1, Bsm1, CYP24A1	2919/2313	No association	(70)

CCS, case-control study; PCCS, population-based case-control study; NCCS, nested case-control study.

**Table II.** Fok1 and renal cancer.

Population studied	Study type	Polymorphism	Cases/controls	Results	Refs.
Central and Eastern European	CCS	Fok1, Bsm1, Taq1	925/1192	The f allele associated with renal cancer risk	(75)
Indian	CCS	Fok1, Bsm1	196/250	The ff genotype associated with high renal cancer risk	(76)

CCS, case-control study.

**Table III.** Bsm1 and breast cancer.

Population studied	Study type	Polymorphism	Cases/controls	Results	Refs.
Caucasian	CCS	Bsm1, Fok1	313/410	The bb genotype is associated with high breast cancer risk	(77)
Caucasian	CCS	Bsm1, Fok1, poly(A)	398/427	Breast cancer occurrence among women with the bb genotype is twice higher than among women with the BB or Bb genotypes	(68)
Caucasian, African-American	PCCS	Bsm1, poly(A)	1631/1435	Postmenopausal Caucasian women with the bb genotype are at higher risk of developing breast cancer	(79)
Caucasian	CCS	Bsm1, Fok1	1180/1547	No association	(73)
Caucasian	CCS	Bsm1, Fok1, poly(A)	181/241	Bsm1 is LD with poly(A) and is associated with breast cancer	(67)
Caucasian	CCS	Bsm1	179/179	Low level of 25(OH)D and the bb genotype increase breast cancer susceptibility	(12)
French Canadian population	CCS	Bsm1, Fok1	Two independent study (225/463 and 622/974)	Bb + bb was associated with slightly increased risk of breast cancer in both studies	(78)
Italian	CCS	Bsm1	88/167	Women with homozygous bb are at higher risk of developing metastases of breast cancer than BB women	(80)
Turkish	CCS	Bsm1, Taq1	78/27	No association	(82)
Caucasian	NCCS	Bsm1, Fok1, Taq1, Apa1, poly(A)	500/500	No association	(71)
Taiwanese	CCS	Bsm1, Taq1, Apa1	80/169	The B allele is associated with breast cancer	(81)
Latinas	CCS	Bsm1, Fok1, poly(A)	143/500	The bb genotype is associated with higher breast cancer risk than BB and Bb	(69)
Chinese	PCCS	Fok1, Bsm1, CYP24A1	2919/2313	No association	(70)

CCS, case-control study; PCCS, population-based case-control study; NCCS, nested case-control study; LD, linkage disequilibrium.

**Table IV.** Bsm1 and renal cancer.

Population studied	Study type	Polymorphism	Cases/controls	Results	Refs.
Japanese	CCS	Bsm1, Apa1, Taq1	135/150	Bsm1 variant not found to be associated with renal cancer	(36)
Central and Eastern European	CCS	Bsm1, Fok1, Taq1	925/1192	The BB genotype is associated with lower risk of renal cancer occurrence	(75)
Indian	CCS	Bsm1, Fok1	196/250	The bb genotype is associated with lower risk of renal cancer	(76)

CCS, case-control study.

**Table V.** Taq1 and breast cancer.

Population studied	Study type	Polymorphism	Cases/controls	Results	Refs.
Hispanic, non-Hispanic	CCS	Taq1, Fok1, Bgl1	814/910	No association	(20)
Caucasian	NCCS	Taq1, Fok1, Bsm1, Apa1, poly(A)	500/500	Women with the TT genotype consuming 902 mg/day calcium have lower risk	(71)
Caucasian	CCS	Taq1	951/627	No association	(83)
Turkish	CCS	Taq1, Bsm1	78/27	Taq1 polymorphism in VDR gene is not linked to breast cancer susceptibility	(84)
Taiwanese	CCS	Taq1, Bsm1, Apa1	80/169	No association	(81)
Finnish	CCS	Taq1, Apa1	483/482	The T allele is associated with lower breast cancer incidence	(86)
German	PCCS	Taq1, Fok1, Cdx2, VDR-5132	1408/2612	In estrogen receptor positive tumor, the t allele is linked to increased risk of breast cancer	(34)
Australian	CCS	Taq1, Fok1, Apa1	135/410	The TT genotype is associated with breast cancer	(72)
Swedish	CCS	Taq1	111/130	The tt genotype is associated with prolonged survival in breast cancer patients	(87)
Indian	CCS	Taq1, Apa1, Poly(A)	160/140	No association	(85)

CCS, case-control study, PCCS; population-based case-control study; NCCS, nested case-control study; VDR, vitamin D receptor.

**Table VI.** Taq1 and renal cancer.

Population studied	Study type	Polymorphism	Cases/controls	Results	Refs.
Central and Eastern European	CCS	Taq1, Bsm1, Fok1	925/1192	Lower risk is associated with the Taq1 t variant	(75)
Japanese	CCS	Taq1	102/204	The TT genotype is more frequent in renal cancer patients	(88)
Japanese	CCS	Taq1, Bsm1, Apa1	135/150	No association	(36)

CCS, case-control study.

**Table VII.** Apa1 and breast cancer.

Population studied	Study type	Polymorphism	Cases/controls	Results	Refs.
Australian	CCS	Apa1, Fok1, Taq1	135/410	The Aa and aa genotypes are associated with breast cancer risk	(72)
Taiwanese	CCS	Apa1, Bsm1, Taq1	80/169	The AA genotype is associated with breast cancer risk	(81)
Finnish	CCS	Apa1, Taq1, Apa1	483/482	Variant ‘a’ genotype is linked to lower risk	(86)
Caucasian	NCCS	Apa1, Bsm1, Fok1, Taq1, poly(A)	500/500	No association	(71)
Indian	CCS	Apa1, Taq1, poly(A)	160/140	No association	(85)

CCS, case-control study; PCCS, population-based case-control study; NCCS, nested case-control study.

**Table VIII.** Apa1 and renal cancer.

Population studied	Study type	Polymorphism	Cases/controls	Results	Refs.
Japanese	CCS	Taq1, Bsm1, Apa1	135/150	AA genotype is frequent in renal cancer cases	(36)

CCS, case-control study.

**Table IX.** Poly(A), Cdx2, Bgl1 and breast cancer.

Population studied	Study type	Polymorphism	Cases/controls	Results	Refs.
Swedish	PCCS	Poly(A)	1502/1510	The SS genotype is associated with breast cancer	(89)
Latinas	CCS	Poly(A), Bsm1, Fok1	143/500	Two SS allele of the poly(A) polymorphism are associated with breast cancer risk	(69)
Caucasian	CCS	Poly(A), Bsm1, Fok1	181/241	The poly(A) L variant is in LD with Bsm1 and is associated with breast cancer	(67)
Caucasian	CCS	Poly(A), Bsm1, Fok1	398/427	The poly(A) LL genotype is associated with breast cancer	(68)
Caucasian	NCCS	Poly(A), Bsm1, Fok1, Apa1, Taq1	500/500	Poly(A) LL positive women can decrease breast cancer risk by consuming 902 mg calcium/day	(71)
Caucasian, African-American	PCCS	Poly(A), Bsm1	1631/1435	No association with poly(A)	(79)
Indian	CCS	Poly(A), Apa1, Taq1	160/140	The long poly(A) L allele is associated with breast cancer	(85)
German	PCCS	Cdx2, Fok1, Taq1, VDR-5132	1408/2612	Cdx2 is not associated with breast cancer	(34)
Hispanic, non-Hispanic	CCS	Bgl1, Fok1, Taq1	814/910	The Bgl1 BB genotype is associated with the decrease of breast cancer development in a group of women with medium pigmentation	(20)

CCS, case-control study; PCCS, population-based case-control study; NCCS, nested case-control study; LD, linkage disequilibrium.

## Abbreviations:

CCS: case-control study;
PCCS: population-based case-control study;
NCCS: nested case-control study;
OR: odds ratio;
UV-B: ultraviolet radiation;
VDR: vitamin D receptor;
LD: linkage disequilibrium;
Bsm1 alleles: (B, b);
poly(A) alleles: [L (long), S (short)];
Apa1 alleles: (A, a);
Taq1 alleles: (T, t);
Fok1 alleles: (F, f);
RFLP: restriction fragment length polymorphism
